# Inequalities in Psychiatric Service Use and Mortality by Migrant Status Following a First Diagnosis of Psychotic Disorder: A Swedish Cohort Study of 1.3M People

**DOI:** 10.1093/schizbullopen/sgab009

**Published:** 2021-03-15

**Authors:** Dafni Katsampa, Syeda F Akther, Anna-Clara Hollander, Henrik Dal, Christina Dalman, James B Kirkbride

**Affiliations:** 1 PsyLife group, Division of Psychiatry, University College London, London, UK; 2 EPICSS, Department of Global Mental Health, Karolinska Institutet, Stockholm, Sweden

**Keywords:** schizophrenia, mortality, inequalities, service utilization, longitudinal, register

## Abstract

It is unclear whether inequalities in mental healthcare and mortality following the onset of psychosis exist by migrant status and region-of-origin. We investigated whether (1) mortality (including by major causes of death); (2) first admission type (inpatient or outpatient); (3) in-patient length of stay (LOS) at first diagnosis for psychotic disorder presentation, and; (4) time-to-readmission for psychotic disorder differed for refugees, non-refugee migrants, and by region-of-origin. We established a cohort of 1 335 192 people born 1984–1997 and living in Sweden from January 1, 1998, followed from their 14th birthday or arrival to Sweden, until death, emigration, or December 31, 2016. People with ICD-10 psychotic disorder (F20–33; *N* = 9399) were 6.7 (95% confidence interval [95%CI]: 5.9–7.6) times more likely to die than the general population, but this did not vary by migrant status (*P* = .15) or region-of-origin (*P* = .31). This mortality gap was most pronounced for suicide (adjusted hazard ratio [aHR]: 12.2; 95% CI: 10.4–14.4), but persisted for deaths from other external (aHR: 5.1; 95%CI: 4.0–6.4) and natural causes (aHR: 2.3; 95%CI: 1.6–3.3). Non-refugee (adjusted odds ratio [aOR]: 1.4, 95%CI: 1.2–1.6) and refugee migrants (aOR: 1.4, 95%CI: 1.1–1.8) were more likely to receive inpatient care at first diagnosis. No differences in in-patient LOS at first diagnosis were observed by migrant status. Sub-Saharan African migrants with psychotic disorder were readmitted more quickly than their Swedish-born counterparts (adjusted sub-hazard ratio [sHR]: 1.2; 95%CI: 1.1–1.4). Our findings highlight the need to understand the drivers of disparities in psychosis treatment and the mortality gap experienced by all people with disorder, irrespective of migrant status or region-of-origin.

## Background

Psychotic disorders affect approximately 3.5% of the population,^1^ and have been associated with poor physical health outcomes, including weight gain, type II diabetes, cardiometabolic diseases, and coronary heart disease.^2–4^ These are likely to contribute to premature mortality,^5^ culminating in reduced life expectancy of approximately 10 to 25 years compared with the general population.^6,7^ This mortality gap also appears to be widening.^8^ These physical health problems are compounded by recurring mental health problems. For example, although more than half of those who experience a first episode of psychosis (FEP) meet criteria for clinical remission within a year of commencing treatment, recent evidence indicates that nearly 80% of patients experience relapse thereafter.^9–11^

Given strong evidence that migrants and their children are on average 2 times more likely to develop a psychotic disorder than the majority population these inequalities may disproportionately affect migrant and minority ethnic communities.^12^ This risk may be even more pronounced in some groups, including people from visible minority backgrounds,^12^ and refugees^13^ who were 3 times more likely to be diagnosed with non-affective psychotic disorder than the native Swedish-born population. In the United Kingdom, there is evidence that people from Black, Asian, and minority ethnic backgrounds with FEP also experience greater excess mortality than the general population.^14,15^ There is also strong evidence from the United Kingdom that people with FEP from some ethnic minority backgrounds—most notably from Black ethnic backgrounds—are more likely to be compulsorily admitted or readmitted for care for any psychiatric disorder.^16^ There is more mixed evidence about ethnic disparities in length of stay (LOS) for inpatient stays following FEP in the United Kingdom. Two studies suggest that several ethnic groups,^17^ including people of Black ethnic origin^17,18^ experienced longer LOS than White participants, and that LOS was longer for people diagnosed with psychosis than other conditions.^17^ By contrast, another study reported a reduction in inpatient stays in Black Caribbean compared with White FEP participants receiving intensive community treatment.^19^ There is also some evidence that people of Black and Asian ethnic origins in the United Kingdom were more and less likely, respectively, to be readmitted following FEP than their White counterparts.^20^

Outside of the United Kingdom, these issues have received less attention, with more mixed evidence available.^21^ For example, no previous studies have examined whether the mortality gap between people with FEP and the general population varies by migrant status, although migrants in general population samples appear to have lower mortality rates than native-born populations,^22,23^ and migrants with FEP appear less likely to die by suicide,^21^ as found in the general population.^24^ Recent evidence from Sweden has found that several migrant groups were at elevated risk of compulsory admission compared with Swedish-born individuals,^25^ supporting UK findings. With respect to readmission, data from New Zealand supports higher readmission rates following a psychiatric diagnosis,^26^ including FEP,^27^ for people of Maori ethnicity compared with the White majority group, while another Canadian study found no evidence that ethnicity was a predictor of admission after a FEP diagnosis.^28^ In Switzerland, one study found that immigrants had lower readmission rates than their Swiss-born counterparts,^29^ but another recent study from the same country suggested higher relapse rates and worse symptomatology amongst migrants who had also experienced adversity.^30^ In the United States, another study reported that African-American participants with psychotic disorders had shorter LOS than White participants, while Asian and Native American participants experienced longer LOS.^31^ No differences in LOS by migrant status were reported in a study in Italy.^32^

To our knowledge, no study has examined disparities in multiple outcomes following FEP—including mortality, type of admission (inpatient or outpatient), LOS or readmission risk—by migrant status in a single study. Understanding these issues would provide vital information for service planners and public health to reduce potential inequalities in psychosis care. We investigated these issues in a cohort of people with a first diagnosis of non-organic psychotic disorder in Sweden, using national register data. Sweden provides a highly suitable setting to investigate these issues. It has a universal healthcare system, including for psychiatric care, free at the point of access for all residents, including refugees. As an European Union (EU) member state, citizens of all EU countries are entitled to work and live in Sweden. In addition, Sweden provides asylum to a large number of refugees and their families from many regions, particularly East Africa, Iraq, Iran, Afghanistan, and countries which were part of the former Yugoslavia (Bosnia and Herzegovina, Croatia, Macedonia, Montenegro, Serbia, and Slovenia). Before 2016, Sweden received more refugees than any other EU county on a per capita basis. We hypothesized that compared with the Swedish-born population with a psychotic disorder, refugee and non-refugee migrants with a psychotic disorder would be more likely to (1) receive inpatient hospital care at first presentation; (2) have longer inpatient care at first admission; (3) be readmitted more quickly after their first recorded diagnosis; and (4) face similar mortality gaps to their Swedish-born counterparts. Consistent with evidence elsewhere on ethnic disparities in mental healthcare, we hypothesized that where these inequalities existed, they would be greater for refugee and non-refugee migrants from regions-of-origin where participants were more likely to be of visible minority status in Sweden (ie, Africa, the Middle East, and Asia).

## Methods

### Sample

Our initial sample was extracted from Psychiatry Sweden, a large, longitudinal database of several linked national Swedish registers containing anonymized data on all people in Sweden granted permanent residency. We used the Register of the Total Population (RTP) to define an initial cohort of people, born between 1984 and 1997, alive and living in Sweden on their 14th birthday, or date of arrival in Sweden, if later. Our cohort was restricted to people born in Sweden to 2 Swedish-born parents, or refugee and non-refugee migrants (born overseas) who arrived in Sweden from January 1, 1998, from regions of origin (Asia, Eastern Europe including Russia, the Middle East & North Africa, Sub-Saharan Africa) with greater than 1000 refugees in the RTP to ensure valid comparisons (Supplementary Table 1), consistent with our previous approach.^13,33^ Migrants who arrived before 1998 were excluded because data on refugee status was not routinely available before this time. We also excluded undocumented or temporary migrants, and people born in Sweden to at least one foreign-born parent from this study.

### Outcomes

We included 4 outcomes in our study: (1) all-cause mortality as recorded in the Causes of Death Register for people who did and did not receive a first diagnosis of International Classification of Diseases (10th revision) (ICD-10) non-affective or affective psychotic disorder (F20–33) recorded in the National Patient Register (NPR). In post hoc analyses, we also examined these mortality gaps for major cause-specific deaths relevant to this cohort, recorded as the underlying causes of death in the register, including (a) deaths by suicide (X60–84) and undetermined intent (Y10–34), (b) deaths due to other external causes (all other ICD-10 Chapter 20, codes beginning V, W, X, and Y), and (c) natural causes (causes of death defined according to ICD-10 Chapters 1–18). In a cohort restricted to people diagnosed with psychotic disorder, we also included the following outcomes, (2) admission type at first presentation (inpatient or outpatient) as recorded in the NPR, (3) time-to-readmission for psychotic disorder, and (4) for participants admitted for in-patient care at first diagnosis, LOS in days.

For all-cause and cause-specific mortality, we followed the whole sample from their 14th birthday or date of immigration if later, until date of death, emigration or December 31, 2016, whichever was sooner. For first admission type, if a participant had both in patient and outpatient records for the same day, we recorded this as an inpatient contact. For readmission, we followed participants from date of first diagnosis until a second contact in the NPR for psychotic disorder, emigration, death, or December 31, 2016, whichever was sooner. We defined a second contact (ie, time-to-readmission) as a second recorded diagnosis of psychotic disorder in the NPR more than 28 days after the index diagnosis, to minimize the possibility that a second diagnostic date recorded in the NPR was part of a participant’s treatment for their first diagnosis. This cut-off was chosen based on local clinical and register expertise (CD, HD). Finally, to estimate inpatient LOS at first diagnosis, we estimated the days between the inpatient date and discharge date recorded in the NPR.

### Exposures

For mortality as an outcome, our primary exposure was a diagnosis of psychotic disorder. For other outcomes around psychiatric care use, our primary exposure was migrant status, defined as people (1) born in Sweden to 2 Swedish-born parents (henceforth, the “Swedish-born population”); (2) people granted asylum under Swedish law and meeting the UNHCR definition of refugee status due to threat of persecution, conflict or other humanitarian crises (UNHCR, 2011) (henceforth refugee migrants); and (3) people born outside of Sweden and permanently settled in Sweden without refugee status (henceforth, “non-refugee migrants”) from one of the regions-of-origin stated above. As a secondary exposure, we classified participants by region-of-origin, as defined above and in Supplementary Table 1. Migrant status and region-of-origin were considered as effect modifiers of the association between psychotic disorder and mortality.

### Confounders

We controlled for sex, age, disposable income and urban residency at cohort entry (age 14 or date of first immigration to Sweden, if later). For mortality and readmission as outcomes, age was treated as time-varying covariate in survival analyses (see below). For other outcomes, age group at diagnosis of psychotic disorder was used. For both definitions of age, we categorized age into the following groups (14–17, 18–21, 22–25, 26–29, and 30–33 years).

Individualized disposable income was estimated from data in the longitudinal integration database for health insurance and labor market studies (LISA) register. Individualized disposable income was based on total annual family income, including benefits, pensions and salary/wages, weighted for household size and composition (ie, dependents). The earliest age at which this was recorded in the LISA register was 16 years, or on arrival in Sweden if later. We classified disposable income into quintiles for the relevant calendar year, relative to the total population in Sweden, to control for inflation. Urban residency was measured as population density at age 14 years old or immigration to Sweden, if later, based on each individual’s Small Area Marketing Statistics (SAMS) area of residence. SAMS are small area geographical units created by Statistics Sweden of 1000–2000, based on election districts in more rural areas, and by dividing cities into socioeconomically homogenous neighborhoods.^34^ We estimated population density from the RTP in each year, divided by the SAMS area (in square kilometers) to give the total population density (people per square kilometer [ppskm]), which we treated as a continuous variable transformed onto the natural logarithm scale given strong positive skew. For all outcomes restricted to people diagnosed with a psychotic disorder (admission type, LOS, time-to-readmission), we also controlled for diagnosis (ICD-10 non-affective [F20–29] vs affective [F30–33] psychotic disorder).

### Statistical Analyses

#### Mortality Outcomes.

First, for the whole cohort, we presented descriptive statistics including crude all-cause mortality rates by migrant status, region-of-origin and diagnosis of psychotic disorder. Second, we fitted Competing Risks Regression (CRR) models to estimate unadjusted and adjusted sub-hazard ratios (sHRs) and 95% confidence interval (95%CIs) to estimate the all-cause mortality gap between the general population and participants diagnosed with psychotic disorder. Third, we tested whether this mortality gap differed by migrant status or region-of-origin, tested via likelihood ratio tests (LRTs) of models with and without the relevant interaction term(s). We repeated these analyses for cause-specific mortality gaps for people with psychotic disorder, and considered whether these differed by migrant status. We fitted cause-specific models using less computationally-intensive Cox proportional hazards models instead of CRR, as evidence from our all-cause mortality analyses suggested the 2 gave comparable point estimates of risk (Supplemental Table 2).

#### Time-to-readmission, Admission Type, and LOS.

We modeled time-to-readmission in the subsample of the cohort diagnosed with psychotic disorder during the follow-up period. We repeated the analytical procedure for mortality, including use of CRR, which we considered to be more appropriate than more conventional Cox proportional hazards survival analysis for both mortality and readmission outcomes, since emigration may have been a plausible competing risk for each. We analyzed the association between admission type at first diagnosis of psychotic disorder (inpatient vs outpatient) and migrant status or region-of-origin using logistic regression, and LOS as an outcome (restricted to the in-patient sample) using linear regression.

For all outcomes, we conducted univariable and multivariable analyses, treating all confounders as *a priori*. We restricted analyses to complete case samples, with missingness explored descriptively. Finally, because migrant status and region-of-origin had the same reference category (ie, Swedish-born) we could not mutually adjust our models for both variables; to ascertain which exposure fitted the data better for each outcome, we inspected Akaike’s Information Criterion (AIC) scores in the final multivariable model fitted with each exposure, where lower score indicated better model fit having penalized for model complexity. All analyses were performed using Stata, version 15.

### Ethical Approval

This study was approved by the Stockholm Regional Ethical Review Board (2010/1185-31/5).

## Results

### Sample Characteristics

We identified 1 335 192 participants who met our inclusion criteria, including 133 362 non-refugee migrants (10.0%) and 24 117 refugees (1.8%) (table 1). Over the follow-up period, 5472 (0.4%) people in this cohort died, with evidence this proportion was lower (0.2%; *P* < .001) for refugee and non-refugee migrants. The sample also included 9399 people diagnosed for the first time with a psychotic disorder during follow-up (0.7%), which varied by migrant status (0.7% to 1.3%; *P* < .001) and was highest for refugees. A higher proportion of non-refugee (52.9%) and refugee (54.2%) migrants were admitted as in-patients for their first diagnosis of psychotic disorder compared with the Swedish-born population (44.0%; *P* < .001), but we observed no differences in median LOS (12 days; interquartile range [IQR]: 3–31; *P* = .85) or proportion of participants readmitted (64.4%; *P* = .16) by migrant status. Participants from Sub-Saharan African made up the lowest proportion of non-refugee migrants (11.6%), but the second-highest proportion of refugee migrants (36.8%), while participants from the Middle East made up the highest proportion of both non-refugee (32.8%) and refugee (37.6%) migrants. Refugee migrants were more likely to be men (57.7%) than in the Swedish-born (51.3%) or non-refugee migrant (49.4%) samples (*P* < .001), and a higher proportion of non-refugee (93.5%) and refugee (97.4%) migrants belonged to the lowest income quintile group compared with Swedish-born participants (57.2%; *P* < .001). Median levels of population density were similar for Swedish-born and refugee participants, but notably higher for non-refugee migrants (*P* < .001; table 1).

**Table 1.** Sample Characteristics by Migrant Status

	Swedish-Born		Non-refugee Migrant		Refugee Migrant		Descriptive Test
	*N*	%	*N*	%	*N*	%	Score (*df*); *P* value^b^
Total^a^	1 177 713	88.2	133 362	10.0	24 117	1.8	-
Died	5141	0.4	280	0.2	51	0.2	174.3 (2); <.001
Psychotic disorder	8054	0.7	1044	0.8	301	1.3	120.8 (2); <.001
In-patient admission	3547	44.0	552	52.9	163	54.2	38.8 (2); <.001
Median LOS [IQR] (days)	12	[3–30]	13	[3–31]	14	[2–39]	0.3 (2); .85^c^
Readmitted	5166	64.1	700	67.1	190	63.1	3.6 (2); .16
Region-of-origin							-
Sweden	1 177 713	100.0	-	-	-	-	
Sub-Saharan Africa	-	-	15 552	11.6	8864	36.8	
Asia	-	-	35 289	26.5	4903	20.3	
Eastern Europe	-	-	38 797	29.1	1295	5.4	
Middle East	-	-	43 754	32.8	9055	37.6	
Age group at cohort exit							11 248.6 (8); <.001
14–17	4741	0.4	2694	2.0	254	1.1	
18–21	235 478	20.0	22 824	17.1	4405	18.3	
22–25	374 192	31.8	33 673	25.3	7202	29.9	
26–29	346 695	29.4	39 943	30.0	6691	27.7	
30–33	216 607	18.4	34 228	25.7	5565	23.1	
Sex							573.4 (2); <.001
Male	604 428	51.3	65 928	49.4	13 903	57.7	
Female	573 285	48.7	67 434	50.6	10 214	42.4	
Diagnosis							23.7 (2); <.001
Non-affective psychosis	5901	73.3	825	79.0	244	81.1	
Affective psychosis	2153	26.7	219	21.0	57	18.9	
Disposable income quintile							62 377.6 (8); <.001
1 (lowest)	594 615	57.2	97 106	93.5	17 380	97.4	
2	267 800	25.8	4221	4.1	348	2.0	
3	109 628	10.6	1556	1.5	83	0.5	
4	42 615	4.1	687	0.7	32	0.2	
5 (highest)	24 342	2.3	255	0.3	5	0.03	
Median population density [IQR] (ppskm)	63.2	[24.9–135.7]	138.1	[59.1–1139.9]	59.1	[19.4–153.0]	44 171.3 (2); <.001^c^

*Note*: df, degrees of freedom; LOS, length of stay; IQR, interquartile range; ppskm, people per square kilometer.

^a^Row percentage.

^b^ *Χ* ^2^-tests with degrees of freedom (df) and *P*-value, unless otherwise stated.

^c^Kruskal-Wallis test.

### All-cause Mortality

The crude mortality rate was higher in people diagnosed with non-affective psychosis (226.4 per 100 000 person-year; 95%CI: 201.0–255.0) than in the remainder of the population (34.4; 95%CI: 33.5–35.3; table 2). Further inspection of these mortality patterns suggested this mortality gap was present for all groups irrespective of migrant status and region-of-origin, with a suggestion that the crude difference in mortality rates between those with and without psychotic disorder was slightly more pronounced in refugees (303.5 vs 23.0 per 100 000 person-years) and Swedish-born participants (237.2 vs 35.2) than non-refugee migrants (96.3 vs 26.2).

**Table 2.** Crude Mortality Rates Per 100 000 Person-Years and sHR Estimates for Mortality in Those With and Without a Diagnosis of Psychotic Disorder, by Migrant Status and Region-of-origin

	Deaths in Psychosis Sample				Deaths in Population-at-risk					
	*N*	%	Person-years at-risk (%)	Mortality Rate^a^ (95%CI)	*N*	%	Person-years at-risk (%)	Mortality Rate^a^ (95%CI)	Unadjusted sHR (95%CI)	Adjusted^b^ sHR (95%CI)
Total	271	100.0	119 704 (100.0)	226.4 (201.0, 255.0)	5201	100.0	15 121 481 (100.0)	34.4 (33.5, 35.3)	6.58 (5.83, 7.43)	6.70 (5.93, 7.58)c
*AIC*									149 314.8	128 863.2
Migrant status										
Swedish-born	253	93.4	106 680 (89.1)	237.2 (209.7, 268.3)	4888	94.0	13 905 193 (92.0)	35.2 (34.2, 36.2)	6.64 (5.86, 7.53)	6.75 (5.95, 7.66)
Non-refugee migrants	10	3.7	10 388 (8.7)	96.3 (51.8, 178.9)	270	5.2	1 029 463 (6.8)	26.2 (23.3, 29.5)	4.36 (2.33, 8.19)	4.86 (2.58, 9.14)
Refugee migrants	8	3.0	2636 (2.2)	303.5 (151.8, 606.8)	43	0.8	186 825 (1.2)	23.0 (17.1, 31.0)	13.39 (6.32, 28.35)	14.06 (6.23, 31.74)
*LRT for interaction* ^d^									*4.8 (2); 0.09*	*3.8 (2); 0.15*
*AIC*									*149* *269.5*	*128* *863.4*
Region-of-origin										
Sweden	253	93.4	106 680 (89.1)	237.2 (209.7, 268.3)	4888	94.0	13 905 193 (92.0)	35.2 (34.2, 36.2)	6.64 (5.86, 7.53)	6.75 (5.95, 7.66)
Sub-Saharan Africa	4	1.5	3500 (2.9)	114.3 (42.9, 304.5)	62	1.2	185 108 (1.2)	33.5 (26.1, 43.0)	3.73 (1.35, 10.27)	2.76 (0.86, 8.88)
Asia	3	1.1	2373 (2.0)	126.4 (40.8, 392.1)	54	1.0	282 389 (1.9)	19.1 (14.6, 25.0)	8.58 (2.72, 27.14)	12.88 (4.01, 41.34)
Eastern Europe	3	1.1	2827 (2.4)	106.1 (34.2, 329.1)	86	1.7	307 371 (2.0)	28.0 (22.6, 34.6)	4.48 (1.42, 14.13)	4.85 (1.54, 15.33)
Middle East	8)	3.0	4325 (3.6)	185.0 (92.5, 369.9)	111	2.1	441 420 (2.9)	25.1 (20.9, 30.3)	8.07 (3.95, 16.49)	8.81 (4.28, 18.14)
*LRT for interaction* ^c^									*2.4 (4); 0.66*	*4.8 (4); 0.31*
*AIC*									*149* *266.4*	***128*** ***840.6***

*Note:* FEP, First episode psychosis; HR, Hazard ratio; 95%CI, 95% confidence interval; LRT, likelihood ratio test; df, degrees of freedom; AIC, Akaike’s information criterion (with differences greater than ~5 points highlighted in bold and indicative of meaningful model improvement, where lower scores favor the improved model).

^a^Per 100 000 person-years.

^b^Adjusted for current age, sex, income quintile, and population density.

^c^From the model without an interaction term between FEP and migrant status.

^d^ *Χ* ^2^ (degrees of freedom); *P*-value.

Following CRR, mortality rates were 6.70 times higher (95%CI: 5.93–7.58) for people diagnosed with psychotic disorder than the remainder of the population, after adjusting for current age, sex, income quintile, and population density (table 2). Although the sHR for this estimate varied from 4.86 (95%CI: 2.58–9.14) for non-refugee migrants to 14.06 (95%CI: 6.23–31.74) for refugees, there was only weak evidence of effect modification by migrant status (LRT *P* = .15) and no evidence of such interaction by region-of-origin (LRT *P* = .31). These findings were in partial contrast to the results of the same models run using Cox proportional hazards modeling, where the presence of effect modification between psychotic disorder and migrant status on mortality would have been interpreted as conventionally meeting statistical significance (ie, adjusted model LRT *P* = .03; Supplementary Table 2).

### Cause-Specific Mortality

Of the 5472 deaths in this cohort up to aged 33 years old, primary causes of death in this cohort were distributed relatively evenly between suicide and undetermined intent (32.2%), other external causes (35.5%) and natural causes (32.8%), although the former outcome was more common amongst the Swedish-born group (32.2%) than non-refugee (24.3%) and refugee (25.5%) migrants, who were more likely to die from natural causes (43.6 and 43.1%, respectively; X^2^*P*-value for cause of death by migrant status on 4 degrees of freedom: 19.4(4); *P* = .001). People diagnosed with psychotic disorder were more likely to die from each specific cause than the population at-risk, but adjusted hazard ratio [aHRs] varied from 2.3 (95% CI: 1.6–3.4) for natural causes to 5.1 (4.0, 6.7) for other external causes and 12.2 (10.4, 14.4) for deaths by suicide or of undetermined intent. There was no statistically robust evidence that these patterns differed by migrant status (table 3).

**Table 3.** Hazard Ratios for Mortality by Major Cause of Death, in People With a Diagnosis of Psychotic Disorder Compared to the General Population, by Migrant Status

	Total Deaths		
Broad Cause of Death	*N* (%)^a^	Unadjusted HR (95%CI)	Adjusted^b^ HR (95%CI)
*Suicide and undetermined intent*			
Overall	1734 (31.7)	12.4 (10.6, 14.6)	12.2 (10.4, 14.4)
Migrant status			
Swedish-born	1653 (32.2)	12.5 (10.5, 14.7)	12.1 (10.2, 14.3)
Non-refugee migrants	68 (24.3)	9.9 (4.4, 21.7)	10.2 (4.6, 22.6)
Refugee migrants	13 (25.5)	40.6 (13.3, 124.1)	32.8 (9.6, 112.1)
*LRT for interaction (Χ* ^*2*^ *(df); P-value)*		*4.1 (2); 0.13*	*2.4 (2); 0.30*
*Other external causes*			
Overall	1945 (35.5)	5.1 (4.1, 6.4)	5.1 (4.0, 6.4)
Migrant status			
** **Swedish-born	1839 (35.8)	5.4 (4.3, 6.8)	5.3 (4.2, 6.7)
** **Non-refugee migrants	90 (32.1)	2.1 (0.5, 8.4)	2.0 (0.5, 8.1)
** **Refugee migrants	16 (31.4)	4.5 (0.6, 34.0)	4.3 (0.6, 32.6)
*LRT for interaction (Χ* ^*2*^ *(df); P-value)*		*2.4 (2); 0.31*	*2.5 (2); 0.28*
*Natural causes*			
Overall	1793 (32.8)	2.1 (1.5, 3.0)	2.3 (1.6, 3.3)
Migrant status			
** **Swedish-born	1649 (32.1)	2.1 (1.4, 3.1)	2.3 (1.6, 3.4)
** **Non-refugee migrants	122 (43.6)	0.8 (0.1, 5.5)	0.9 (0.1, 6.4)
** **Refugee migrants	22 (43.1)	6.8 (1.6, 29.2)	10.5 (2.3, 46.7)
*LRT for interaction (Χ* ^*2*^ *(df); P-value)*		*3.3 (2); 0.20*	*4.0 (2); 0.14*

*Note:* ^a^Column percentages per category (total, Swedish-born, non-refugee migrant, refugee migrant). For any given category %s sum to 100.0%. For example, for overall total deaths, 31.7% were deaths by suicide or undetermined intent, 35.5% other external causes and 32.8% natural causes.

^b^Adjusted for current age, sex, income quintile, and population density.

### Admission Type at First Diagnosis, In-patient LOS and Time-to-readmission

Non-refugee (Odds ratio [OR]: 1.40; 95%CI: 1.21–1.61) and refugee (OR: 1.42; 95%CI: 1.09–1.84) migrants were more likely to be admitted as in-patients at first diagnosis of psychotic disorder, after adjustment for confounders (table 4). Further inspection suggested region-of-origin provided a better fit to this data, with migrants from Sub-Saharan Africa (OR: 2.06; 95%CI: 1.63–2.61) and Asia (OR: 1.48; 95%CI: 1.13–1.96) more likely to be admitted as in-patients than the Swedish-born population, with no evidence of differences in inpatient or outpatient admissions for people from Eastern European or Middle Eastern backgrounds.

**Table 4.** Healthcare Use Following First Diagnosis of Psychotic Disorder, by Migrant Status and Region-of-Origin

	In-patient Sample^a^		In-patient Admission at First Diagnosis		LOS (Days)		Readmission for Psychotic Disorder^a^		Time-to-Readmission		
	*N*	%	Unadjusted OR (95% CI)	Adjusted^b^ OR (95% CI)	Unadjusted Β (95% CI)	Adjusted^b^ Β (95% CI)	*N*	%	Unadjusted sHR (95%CI)	Adjustment 1^c^ sHR (95%CI)	Adjustment 2^d^ sHR (95%CI)
Total	4262	100.0	-	-	-	-	5962	100.0	-	-	-
Migrant status											
Swedish-born	3547	83.2	1	1	1	1	5086	85.3	1	1	1
Non-refugee migrants	552	13.0	1.43 (1.25– 1.62)	1.40 (1.21– 1.61)	0.86 (−6.38– 8.11)	2.32 (−5.74–10.38)	689	11.6	1.14 (1.05– 1.23)	1.09 (1.00– 1.20)*	1.08 (0.99– 1.18)**
Refugee migrants	163	3.8	1.50 (1.19– 1.89)	1.42 (1.09– 1.84)	11.03 (−1.66– 23.71)	13.75 (−0.86– 28.36)***	187	3.1	0.99 (0.85– 1.14)	0.96 (0.82– 1.13)	0.93 (0.79– 1.09)
*AIC*			*12* *915.6*	*12* *075.7*	***48*** ***350.3***	***46*** ***079.2***			*102* *630.6*	*96* *143.4*	*95* *886.3*
Region-of-origin											
Sweden	3547	83.2	1	1	1	1	5086	85.3	1	1	1
Sub-Saharan Africa	237	5.6	2.14 (1.73– 2.64)	2.06 (1.63– 2.61)	5.99 (−4.69– 16.68)	6.96 (−4.85–18.77)	263	4.4	1.26 (1.11– 1.42)	1.24 (1.09– 1.41)	1.19 (1.05– 1.35)
Asia	135	3.2	1.41 (1.10– 1.80)	1.48 (1.13– 1.96)	0.24 (−13.57– 14.06)	2.20 (−12.94–17.35)	161	2.7	1.02 (0.87– 1.20)	0.99 (0.83– 1.18)	0.99 (0.83– 1.18)
Eastern Europe	134	3.1	1.15 (0.91– 1.46)	1.09 (0.84– 1.40)	−0.40 (−14.37- 13.57)	1.93 (−13.36–17.21)	181	3.0	1.12 (0.96– 1.31)	1.09 (0.93– 1.29)	1.05 (0.89– 1.24)
Middle East	209	4.9	1.21 (1.00– 1.47)**	1.16 (0.94– 1.44)	4.22 (−7.03– 15.48)	5.75 (−6.61–18.11)	271	4.6	1.01 (0.90– 1.14)	0.95 (0.84– 1.09)	0.96 (0.84– 1.09)
*AIC*			***12*** ***898.8***	***12*** ***060.8***	*48* *355.6*	*46* *084.7*			*102* *630.4*	***96*** ***140.6***	*95* *887.4*

*Note*: LOS, length of stay; OR, odds ratio; 95% CI, 95% confidence interval; Β, Beta; sHR, sub-Hazard ratio; AIC, Akaike’s information criterion (with differences greater than ~5 points highlighted in bold and indicative of meaningful model improvement, where lower scores favor the improved model).

^a^Total sample with first diagnosis of psychotic disorder: 9399. For analyses on in-patient LOS and time-to-readmission, we excluded 95 participants with errors in their in-patient discharge dates (including a second diagnosis or death recorded before their discharge date from their index admission). Excluded participants were: 81 (85.3%) Swedish-born, 11 (11.6%) non-refugee migrants, 3 refugee migrants (3.2%), similar to the pattern in the included in-patient sample (Fisher’s Exact Test *P* = .95).

^b^Adjusted for age group at diagnosis, sex, income quintile, population density and diagnosis type (non-affective vs affective psychosis).

^c^Adjusted for current age, sex, income quintile, population density and diagnosis type.

^d^Adjusted for current age, sex, income quintile, population density, diagnosis type, first admission type and length of inpatient stay variable.

*P = .04; **P = .08; ***P = .07.

For in-patient LOS and time-to-readmission analyses, we excluded 95 participants with inconsistent inpatient dates, including a second diagnosis or death date recorded before discharge for the index diagnosis. No differences in the excluded sample were found by migrant status (*P* = .95; table 4). No clear differences in LOS were found by migrant status or region-of-origin, although we observed weak evidence of longer in-patient stays in refugees vs Swedish-born participants at first diagnosis for psychotic disorder (13.75 days; 95%CI: −0.86–28.36; *P* = .07).

In multivariable competing risk regression models, time-to-readmission did not differ between refugees and the Swedish-born population (sHR: 0.96; 95%CI: 0.82–1.13) with some evidence of shorter time-to-readmission for non-refugee migrants (sHR: 1.10; 95%CI: 1.00–1.20; *P* = .04), particularly those from Sub-Saharan Africa (sHR: 1.26; 95%CI: 1.11–1.42). After further adjustment for admission type and in-patient LOS at first diagnosis, there was no longer any statistically significant evidence for shorter time-to-readmission for non-refugee migrants (sHR: 1.08; 95% CI: 0.99–1.18; *P* = .08), though such an effect remained evident in migrants from Sub-Saharan Africa (sHR: 1.19; 95% CI: 1.05–1.35). These patterns were similar when fitted under a Cox proportional hazards model (Supplementary Table 3).

## Discussion

### Principal Findings

We investigated whether there were disparities in multiple outcomes following a first diagnosis of a psychotic disorder, by migrant status and region-of-origin, for the first time in a single national cohort. In our young cohort (followed until a maximum of 33 years old), we observed a large (over 6-fold) mortality gap between people diagnosed with a psychotic disorder and the remainder of the population up to 19 years after first diagnosis. This gap was evident for all broad causes of death, and most pronounced for death by suicide or of undetermined intent. Despite some variation in point estimates, there was no formal evidence that this mortality gap varied by migrant status or region-of-origin, though was present for nearly all groups save migrants of Sub-Saharan African origin. Non-refugee and refugee migrants were 40% more likely to be admitted as inpatients at first diagnosis for psychotic disorder, consistent with our earlier work that they were also more likely to be compulsorily detained. Further inspection suggested risk of inpatient admission was greatest for people from Sub-Saharan Africa and Asia in our cohort. Fewer differences in in-patient LOS at first hospitalization for psychotic disorder were observed; the average length of inpatient stay in our cohort was around 12 days. Time-to-readmission for psychotic disorder was shorter for migrants from Sub-Saharan Africa, not explained by confounding factors, including admission type or length of inpatient stay at first diagnosis.

### Meaning of Findings

Consistent with previous evidence,^5,6,15^ we identified a persistent and gross mortality gap between people with and without psychotic disorder in Sweden. This mortality gap was detectable across all groups, irrespective of migrant status or region-of-origin, consistent with findings from 2 studies in South London which found the mortality gap for people with FEP was similar across different ethnic groups.^14,15^ To our knowledge, this is the first study to inspect the mortality gap in refugees with psychotic disorder; although we did not detect statistically significant effect modification between migrant status and psychosis on all-cause or cause-specific mortality, refugees had the largest point estimate for the all-cause mortality gap (over 14 times greater for refugees with psychosis than refugees without psychosis), as well as for deaths by suicide/undetermined intent and natural causes. Larger studies are required to examine this potential disparity, but more immediately, clinicians working in refugee populations with psychotic disorders should be aware of the particularly high crude mortality rates experienced by this group (303.5 deaths per 100 000 people per year). While we have previously shown that migrants are at lower risk of death by suicide in the general population,^24^ this pattern differs for non-refugee and refugee migrants diagnosed with a psychotic disorder, who were, respectively over 10 and 32 times more likely to die by suicide/undetermined intent than their non-psychotic counterparts.

Previous research strongly supports higher rates of psychotic disorder amongst migrants and their children,^12^ including higher first admission rates to hospital where this is the primary mode of care.^35^ Few studies have directly compared whether migrants are more or less likely to receive inpatient vs outpatient care during treatment for their first diagnosis of psychotic disorder. We extended this research to demonstrate that both non-refugee and refugee migrants were around 40% more likely to receive an inpatient admission at first diagnosis compared with their Swedish-born counterparts. Similarly to the United Kingdom,^16^ this risk was greatest for people from likely visible minority migrant backgrounds from Sub-Saharan Africa and Asia. Several factors may explain this finding, including the increased likelihood of compulsory admission for psychiatric care, including FEP, for people from Black, Asian and other ethnic minority backgrounds, which has been strongly and consistently observed in Sweden,^25^ the United Kingdom ,^16^ and elsewhere,^36^ and would typically be accompanied by inpatient rather than outpatient treatment. Greater likelihood of inpatient admission may also be explained by a longer duration of untreated psychosis [DUP] observed in some migrant groups,^37,38^ potentially delaying treatment and resulting in more severe FEP requiring hospitalization. Several factors may delay treatment-seeking in migrant groups, including unfamiliarity of the legal entitlement to healthcare in Sweden, or other cultural or structural barriers in accessing timely treatment, including language barriers, multiple complex health needs requiring treatment or fear of legal repercussions for refugees.^39,40^ We suggest that these findings will generalize to other settings where non-refugee and refugee migrants may be at both greater risk of psychosis,^41^ and face differential outcomes in their treatment pathways.^42^

We also observed tentative evidence, in need of replication in larger samples, that refugees experienced a 2-week longer in-patient admission at first diagnosis for psychosis than their Swedish-born counterparts. This may highlight greater additional needs in this group, and suggests that clinical services in settings with higher refugee populations will require appropriate tailoring to ensure equitable provision of effective and timely care. Unlike studies in the United Kingdom, we found no evidence of longer inpatient stays for people from likely Black backgrounds (ie, in our sample, migrants from Sub-Saharan Africa), or from any other migrant background, though imprecision around our estimates suggests our ability to detect these effects may have been limited by sample size.

We found non-refugee migrants, but not refugees, were readmitted more quickly for psychotic disorder than the Swedish-born group over the follow-up period. This suggests further work is needed to unpack these differential findings, which were not accounted for after adjustment for a priori confounders or type of admission or length of in-patient stay. Refugees may receive additional welfare support by virtue of their legal status compared with non-refugee migrants which protects against readmission, or alternatively may be less likely to re-engage with services for some of the reasons outlined above. Previous studies have identified other inequalities in mental healthcare offered to migrants and refugees, which need to be explored in this context. For example, migrant and ethnic minority groups experiencing psychotic disorder are less likely to be offered newer second-generation antipsychotics,^43,44^ including clozapine^45^ to manage psychotic symptoms, while lower rates of antipsychotic medication adherence have been observed for some ethnic minority groups.^46,47^

### Strengths and Limitations

Our study has a number of methodological strengths, including a large, national cohort design with substantial diversity by migrant status and region-of-origin, and virtually no missing data. Diagnoses recorded in the Swedish National Patient Register have good validity.^48,49^ The register has achieved 100% coverage of all inpatient settings since 1987, and outpatient settings since 2006. We used CRR appropriate for 2 outcomes (mortality, time-to-readmission) as emigration (for both) and death (for time-to-readmission) may have been legitimate competing events; indeed, fitting mortality data using Cox proportional hazards models may have resulted in erroneous interpretation of the results (Supplementary Table 1). Despite our large cohort size, including over 9000 people with a first diagnosis of psychotic disorder, we may have had limited power to detect differences by migrant status or region-of-origin for some outcomes, including cause-specific mortality gaps, LOS and time-to-readmission, as well as our effect modification analyses with respect to mortality. We were unable to control for parental education or family history of psychiatric problems giving limited availability on these covariates in migrant groups recorded in the registers. Information regarding pre-migration experiences was also unavailable. We excluded children of migrants from this study as we were interested in understanding possible inequalities in outcomes after first diagnosis of psychotic disorder for first-generation refugee and non-refugee migrants. Children of migrants continue to face elevated risk of psychosis, in Sweden^50^ and elsewhere,^12^ and may also experience differential outcomes thereafter, which should be considered in further research.

### Implications

Our study highlights complex patterns of disparities by migrant status and region-of-origin in a cohort of young people diagnosed with psychotic disorder for the first time in Sweden. The mortality gap between people with psychosis and the general population appears a ubiquitous disparity irrespective of migrant background, and suggests selected interventions are needed to prevent key causes of reduced life expectancy in this group, including suicide prevention and physical health interventions. Greater likelihood of receiving inpatient vs outpatient care for migrants is consistent with narratives of delayed treatment, complex health needs, and worse (including coercive) pathways to and through care, perhaps culminating in longer inpatient stays for refugees and quicker readmission for migrants of Sub-Saharan African origin. These findings highlight the need to examine and remove barriers to equitable service provision, including the need to design culturally sensitive, early detection and intervention services that offer accessible, destigmatized, and timely treatment for all residents in Sweden irrespective of migrant status or region-of-origin.

## Supplementary Material

sgab009_suppl_Supplementary_Materials
