# Supplementary material for: Inequalities in Psychiatric Service Use and Mortality by Migrant Status Following a First Diagnosis of Psychotic Disorder: A Swedish Cohort Study of 1.3M People
Source: Schizophr Bull Open. 2021 Mar 15;2(1):sgab009. doi: 10.1093/schizbullopen/sgab009 (PMC8052494; doi:10.1093/schizbullopen/sgab009)
Supplement: sgab009_suppl_Supplementary_Materials [file sgab009_suppl_supplementary_materials.docx]

| **Region of origin^1^** | **SMA classification^2^** | **Cohort characteristics (N=1,335,192)** | | | **Countries^3^** |
| --- | --- | --- | --- | --- | --- |
|  |  | Psychotic disorders (%) | Deaths (%) | Participants (%) |  |
| Sub-Saharan Africa | West Africa | 52 (0.6) | 10 (0.2) | 2,680 (0.2) | Benin, Burkina Faso, Cape Verde, Gambia, Ghana, Guinea, Guinea Bissau, Ivory Coast, Liberia, Mali, Mauritania, Niger, Nigeria, Senegal, Sierra Leone, Togo |
|  | East Africa | 245 (2.6) | 41 (0.8) | 16,919 (1.3) | Djibouti, Eritrea, Ethiopia ,Somalia |
|  | Africa, other | 81 (0.9) | 15 (0.3) | 4,787 (0.4) | Angola, Botswana, Burundi, Cameroon, Central African Republic, Chad, Comoros, Congo, Democratic Republic of Congo, Equatorial Guinea, Gabon, Kenya, Lesotho, Madagascar, Malawi, Mauritius, Mozambique, Namibia, Rwanda, Sao Tome and Principe, Seychelles, South Africa, Swaziland, Tanzania, Uganda, Zambia, Zanzibar |
| Asia | Central Asia | 148 (1.6) | 35 (0.6) | 19,569 (1.5) | Afghanistan, Armenia, Azerbaijan, Bangladesh, Bhutan, Georgia, India, Kazakhstan, Kyrgyzstan, Maldives, Nepal, Pakistan, Sri Lanka, Tajikistan, Turkmenistan |
|  | Northeast Asia | 34 (0.4) | - (0.1)^4^ | 8,841 (0.7) | China, Japan, Mongolia, People’s Republic of Korea, South Korea, Taiwan |
|  | Southeast Asia | 74 (0.8) | 18 (0.3) | 10,896 (0.8) | Brunei, Cambodia, East Timor, Hong Kong, Indonesia, Laos, Malaysia, Myanmar, Philippines, Singapore, Thailand, Vietnam |
| Eastern Europe & Russia | Eastern Europe | 110 (1.2) | 35 (0.6) | 18,129 (1.4) | Albania, Belarus, Bulgaria, Czech Republic, Hungary, Moldova, Poland, Romania, Slovakia, Ukraine |
|  | Former Yugoslavia | 103 (1.1) | 33 (0.6) | 13,265 (1.0) | Bosnia Herzegovina, Croatia, Kosovo, Macedonia, Montenegro, Serbia, Slovenia |
|  | Russia & the Baltic States | 69 (0.7) | 21 (0.4) | 8,698 (0.7) | Estonia, Latvia, Lithuania, Russia |
| Middle East & North Africa | Iran | 62 (0.7) | 11 (0.2) | 5,483 (0.4) | Iran |
|  | Iraq | 259 (2.8) | 81 (1.5) | 31,429 (2.4) | Iraq |
|  | Middle East, other | 77 (0.8) | 26 (0.5) | 13,450 (1.0) | Bahrain, Cyprus, Israel, Jordan, Kuwait, Lebanon, Oman, Palestine, Qatar, Saudi Arabia, Syria, United Arab Emirates, Yemen, Turkey |
|  | North Africa | 30 (0.3) | - (0.0)^4^ | 2,447 (0.2) | Algeria, Egypt, Libya, Morocco, Tunisia |

**S1 Table: Region-of-origin classification and basic sample characteristics of migrant groups**

SMA: Swedish Migration Agency; PYAR: Person-years at-risk

^1^Predefined by authors for analysis, based on SMA classification. Sweden not shown.

^2^Categories provided by the Swedish Migration Agency for research purposes, reflecting major migrant and refugee flows to Sweden

^3^Countries of origin, as defined by the Swedish Migration Agency, in each SMA category. Country-level data is not made available by the SMA/Statistics Sweden for research purposes.

^4^Supressed due to small cell sizes

**S2 Mortality gap between people with and without psychotic disorder, by migrant status and region of origin following Cox proportional survival modelling**

|  | **Mortality gap (FEP vs. population at-risk)** | |
| --- | --- | --- |
|  | **Unadjusted HR (95%CI)** | **Adjusted^1^ HR (95%CI)** |
| **Overall** | 6.33 (5.61, 7.16) | 6.50 (5.74, 7.35)^2^ |
| **Migrant status** |  |  |
| Swedish-born | 6.49 (5.72, 7.37) | 6.63 (5.84, 7.53) |
| Non-refugee migrants | 3.35 (1.78, 6.30) | 3.51 (1.86, 6.63) |
| Refugee migrants | 12.46 (5.86, 26.51) | 12.81 (5.65, 29.0) |
| *LRT for interaction (Χ^2^ (df); p-value)* | *7.6 (2); 0.02* | *6.7 (2); 0.03* |
| *AIC* | *148,930.2* | *128,591.1* |
| **Region-of-origin** |  |  |
| Sweden | 6.49 (5.72, 7.37) | 6.63 (5.84, 7.53) |
| Sub-Saharan Africa | 3.15 (1.15, 8.67) | 2.27 (0.70, 7.27) |
| Asia | 5.95 (1.86, 19.02) | 8.09 (2.48, 26.37) |
| Eastern Europe | 3.45 (1.09, 10.91) | 3.47 (1.09, 11.02) |
| Middle East | 6.86 (3.35, 14.06) | 7.37 (3.57, 15.21) |
| *LRT for interaction (Χ^2^ (df); p-value)* | *3.8 (4); 0.44* | *6.0 (4); 0.20* |
| *AIC* | *148,934.3* | ***128,585.0*** |

FEP: First episode psychosis; HR: Hazard ratio; 95%CI: 95% confidence interval; LRT: likelihood ratio test; df: degrees of freedom; AIC: Akaike’s information criterion (with differences greater than ~5 points highlighted in **bold** and indicative of meaningful model improvement, where lower scores favor the improved model)

^1^Adjusted for current age, sex, income quintile, and population density

**S3 Time to re-admission, by migrant status and region of origin following Cox proportional survival modelling**

|  | **Unadjusted HR (95%CI)** | **Adjusted^1^ HR (95%CI)** | **Adjusted^2^ HR (95%CI)** |
| --- | --- | --- | --- |
| **Migrant status** |  |  |  |
| Swedish-born | 1 | 1 | 1 |
| Non-refugee migrants | 1.15 (1.06-1.24) | 1.10 (1.01-1.20) | 1.08 (0.99-1.18) |
| Refugee migrants | 0.99 (0.85-1.14) | 0.96 (0.82-1.13) | 0.93 (0.79-1.09) |
| *AIC* | *104,208.6* | *97,613.8* | *95,757.6* |
| **Region-of-origin** |  |  |  |
| Sweden | 1 | 1 | 1 |
| Sub-Saharan Africa | 1.26 (1.11-1.42) | 1.24 (1.09-1.42) | 1.18 (1.03-1.35) |
| Asia | 1.02 (0.88-1.20) | 0.99 (0.83-1.18) | 1.00 (0.84-1.19) |
| Eastern Europe | 1.15 (0.99-1.33) | 1.12 (0.96-1.31) | 1.07 (0.91-1.25) |
| Middle East | 1.01 (0.90-1.14) | 0.95 (0.83-1.09) | 0.96 (0.84-1.09) |
| *AIC* | *104,208.4* | *97,610.8* | *95,759.0* |

HR: Hazard ratio; 95%CI: 95% confidence interval; AIC: Akaike’s information criterion (with differences greater than ~5 points highlighted in **bold** and indicative of meaningful model improvement, where lower scores favor the improved model)

^1^Adjusted for current age, sex, income quintile, population density and diagnosis type (non-affective versus affective)

^2^Adjusted for current age, sex, income quintile, population density, diagnosis type (non-affective versus affective) and admission type and inpatient length of stay
